# Association of Imaging-based Predictors with Outcome in Different Treatment Options for Intracerebral Hemorrhage

**DOI:** 10.1007/s00062-024-01406-2

**Published:** 2024-04-26

**Authors:** Roger M. Krzyżewski, Borys M. Kwinta, Krzysztof Stachura, Tadeusz J. Popiela, Roman Pułyk, Agnieszka Słowik, Jerzy Gąsowski, Kornelia M. Kliś

**Affiliations:** 1 Department of Neurosurgery and Neurotraumatology, Jagiellonian University Medical College, Jakubowskiego 2 Street, 30-688 Kraków, Poland; 2 Department of Radiology, Jagiellonian University Medical College, Kraków, Poland; 3 Department of Neurology, Jagiellonian University Medical College, Kraków, Poland; 4 Department of Internal Medicine and Gerontology, Jagiellonian University Medical College, Kraków, Poland

**Keywords:** Hemorrhagic stroke, Mortality, Craniotomy, Computational analysis, Surgical treatment

## Abstract

**Purpose:**

Intracerebral hemorrhage is the deadliest form of stroke. This study aimed to enhance the prediction of 30-day mortality in intracerebral hemorrhage patients by integrating computational parameters.

**Methods:**

This study retrospectively analyzed 435 patients with spontaneous intracerebral hemorrhage (ICH). Utilizing the acquired computed tomography (CT) images, we extracted the contour and visual representation of ICH. For the extracted contour, the analysis encompassed factors including compactness, fractal dimension, Fourier factor, and circle factor. For the images depicting ICH, we calculated various factors related to density distribution including mean, coefficient of variance, skewness and kurtosis, as well as texture parameters, such as energy, entropy, contrast and homogeneity. To assess the impact of surgical treatment on 30-day mortality, logistic regression analysis was used.

**Results:**

A total of 126 patients (29.09%) died within 30 days. A total of 62 (14.25%) patients underwent surgical treatment. Multivariate logistic regression analysis revealed that surgical treatment was independently associated with a lower risk of 30-day mortality (odds ratio, OR 0.226, 95% confidence interval, CI 0.049–0.85; *p* = 0.039). Based on the moderated analysis, we found that the volume of ICH (OR 0.905, 95% CI 0.902–0.908; *p* < 0.001) and ICH energy (OR 1.389, 95%CI 0.884–0.988; *p* = 0.010) had positive moderating effect on such associations while the presence of intraventricular blood had negative moderating effect (OR 1.154, 95% CI 1.034–1.628; *p* = 0.010).

**Conclusion:**

Patients exhibiting a higher volume and energy of ICH might benefit from surgical treatment; however, this efficacy was found to be diminished in cases involving the presence of intraventricular blood.

**Supplementary Information:**

The online version of this article (10.1007/s00062-024-01406-2) contains supplementary material, which is available to authorized users.

## Introduction

Spontaneous intracerebral hemorrhage (ICH) accounts for 10–30% of all acute strokes [1] and is characterized by the highest mortality rate compared to all other subtypes [2]. Additionally, it is associated with a high percentage of functional dependence [3]. Therefore, the exploration of potential treatment strategies is currently the subject of extensive research. Particular attention has been given to investigating the potential advantages of surgical interventions in the treatment of ICH. The choice of surgical management in ICH is understood to be associated with several benefits, such as reduction of the mass effect of ICH and minimizing the impact of toxic blood components on brain tissue [4]. Although meta-analyses and reviews suggested a positive impact of surgical treatment on mortality [5], the results of clinical trials in this matter remain inconclusive. The first randomized, controlled trial, surgical treatment for intracerebral hemorrhage (STICH), failed to demonstrate the benefits of surgically removing ICH; however, a subsequent study STICH II, suggested that surgical treatment of lobar hematomas might improve outcomes [7]. Further trials focusing on minimally invasive techniques for ICH removal have also suggested the potential benefit of such procedures [8, 9]. The American Heart Association guidelines state that the efficacy of surgical treatment for ICH is inconclusive and recommended surgical intervention for patients with cerebellar hemorrhage and neurological deterioration or for those suffering from brainstem compression [10]. Predictors of poor outcome following ICH have also been extensively studied. Radiomics analysis of ICH imaging has shown potential correlations with mortality and risk of hemorrhage enlargement [11–13]; however, there is a lack of research exploring the potential association between radiomics analysis and outcomes following surgical treatment of ICH. Consequently, our study aims to re-examine potential predictors of 30-day mortality post-ICH with particular emphasis on the implications of surgical treatment and ICH appearance on computed tomography (CT) imaging.

## Methods

### Study Group

We retrospectively analyzed patients hospitalized in the Department of Neurology or in the Department of Neurosurgery of the University Hospital in Krakow between January 2013 and October 2020, who met the following criteria: (1) had first spontaneous ICH diagnosed based on head CT, (2) presence of intracranial aneurysm or other vascular abnormalities was excluded based on angio-CT or digital subtraction angiography, (3) had no other intracranial pathologies including tumors, (4) had no previous neurosurgical or endovascular procedures and (5) had no history of spontaneous ICH. From the medical records we obtained data such as current diseases and medications, blood tests results taken < 4 h following admission and Glasgow coma scale (GCS) score taken on admission. We also obtained the CT images taken < 4 h after admission. Based on these images we assessed parameters such as ICH location, volume and presence of intraventricular hemorrhage (IVH). For study group we assessed 30-day mortality.

### Computational Analysis of ICH Appearance

**Fig. 1:**
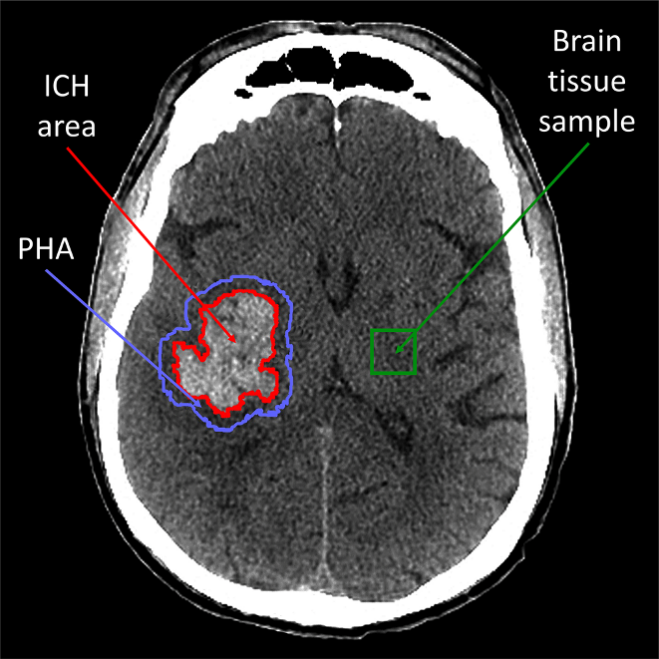
Exemplary axial head CT image segmentation. *Red contour* intracerebral hemorrhage contour with intracerebral hemorrhage image inside; area between *blue and red contour* perihemorrhage area image; area inside *green square* brain tissue sample image. *ICH* intracerebral hemorrhage, *PHA* perihemorrhage area

The authors developed a software written in Python and performed a series of transformations on CT images obtained from each patient. Each image was represented as a two-dimensional pixel array with density values between 0 and 255. Then we semiautomatically extracted the images containing ICH, image of 4 mm thickness brain tissue surrounding ICH, defined as the perihemorrhage area (PHA) and 20 × 20 pixel image of healthy brain tissue for reference (Fig. 1). Additionally, we extracted a curve representing contours of the ICH. For the extracted images of ICH and PHA we analyzed the distribution of density values by calculating its mean, coefficient of variance (mean/standard deviation), skewness and kurtosis. Additionally, we computed these parameters for the brain tissue sample. The presented ICH and PHA density distribution values are normalized to healthy brain tissue. Furthermore, we conducted texture analysis by constructing a gray level co-occurrence matrix (matrix with distribution of co-occurring pixel values at a given offset) [14]:

$$\begin{aligned} &M_{\Updelta x,\Updelta y}\left(i,j\right)=\\&\quad \sum\limits_{x=1}^{n}\sum\limits_{y=1}^{m}\begin{cases} 1,\ \textit{if}\ I\left(x,y\right)=i\ \textit{and}\ I\left(x+\Updelta x,y+\Updelta y\right)=j\\ 0,\ \textit{otherwise} \end{cases}\end{aligned}$$

where *i* and *j* are pixel values, *x* and *y* are the spatial positions in the *n ×* *m *size image *I* and *(∆x, ∆y)* is the given offset. For each image we constructed four matrices with offset d = (2.2) in directions of 0°, 45°, 90° and 135°. Based on matrices we calculated the following descriptors:

$$\text{Energy}=\sqrt{\sum\limits_{i,j=0}^{V-1}\left(M\left(i,j\right)\right)^{2}}$$

$$\text{Homogeneity}=\sum\limits_{i,j=0}^{V-1}\frac{M\left(i,j\right)}{1+\left(i+j\right)^{2}}$$

$$\text{Correlation}=\sum\limits_{i,j=0}^{V-1}M\left(i,j\right)\left[\frac{\left(i-\overline{i}\right)\left(j-\overline{j}\right)}{\sqrt{\left({\sigma }_{i}^{2}\right)\left({\sigma }_{j}^{2}\right)}}\right]$$

$$\text{Contrast}=\sum\limits_{i,j=0}^{V-1}\left(i-j\right)^{2}M\left(i,j\right)$$

For extracted contours we calculated the following parameters: fractal dimension, Fourier factor, circle factor and compactness. Details about contour parameters and used software were presented in our previous research [15].

We used the Shapiro-Wilk test to assess normality. For continuous variables that followed a normal distribution, we utilized the t‑test and for those variables which were not normally distributed, we performed the Mann-Whitney U-test. We used the χ2 test for proportional variables. We expressed continuous variables as mean ± standard deviation. To analyze independent predictors of 30-day mortality after ICH we used multivariate logistic regression analysis. Additionally, we employed logistic regression analysis to assess the factors moderating effect of surgical treatment on 30-day mortality. To identify the cut-off points for moderating the effects of ICH energy and volume, Receiver Operating Characteristic (ROC) curve analysis was utilized. P‑values < 0.05 were considered statistically significant. All statistical analyses and the preparation of figures were conducted using RStudio version 8.5 for Windows (Posit, Boston, MA, USA).

## Results

### Univariate and Multivariate Analyses

A total of 435 patients met the inclusion criteria. Detailed characteristics of studied groups are presented in Table 1. Of the patients 126 (29.09%) died within the first 30 days and 62 patients (14.25%) underwent surgical treatment. Patients who underwent surgical treatment had significantly lower GCS score upon admission (9.11 ± 4.84 vs. 10.81 ± 4.24; *p* < 0.01), significantly less often had hypertension (30.65% vs. 52.02%; *p* < 0.01) and diabetes mellitus (4.84% vs. 17.52%; *p* = 0.01). Patients receiving surgical treatment were also significantly more likely to have ICH in the temporal lobe (22.58% vs. 12.94%; *p* = 0.045) or cerebellum (24.19% vs. 9.97%; *p* < 0.01) and significantly less frequently in the basal ganglia (22.58% vs. 36.93%; *p* = 0.03). Regarding the CT appearance of ICH, these patients exhibited significantly higher compactness (0.66 ± 0.22 vs. 0.58 ± 0.2; *p* < 0.01), energy (0.23 ± 1.31 vs. 0.04 ± 0.93; *p* = 0.048), Contrast (0.31 ± 1.21 vs. 0.05 ± 0.95; *p* < 0.01), and Homogeneity (0.32 ± 1.09 vs. 0.05 ± 0.97; *p* < 0.01), but significantly lower Fourier Factor (0.82 ± 0.09 vs. 0.86 ± 0.07; *p* < 0.01) and Circle Factor (0.35 ± 0.15 vs. 0.41 ± 0.15; *p* < 0.01) (Table 1). Multivariate analysis of possible predictors of 30-day mortality (Table 2), performed for entire study group, revealed that surgical treatment was independently associated with lower 30-day mortality (OR:0.226; 95%CI:0.049–0.85; *p* = 0.039).

**Table 1:** Comparison of analyzed factors between patients who underwent surgical and conservative treatment

Variable	Surgical treatment (*n* = 62)	Conservative treatment (*n* = 373)	*p*-value
Female gender [%, (n)]	3.23 (2)	40.16 (149)	< 0.001
Age (years) ± SD	73.02 ± 17.25	71.79 ± 15.99	0.582
Glasgow Coma Scale ± SD	9.11 ± 4.84	10.81 ± 4.24	0.004
Volume (ml) ± SD	36.86 ± 22.72	32.77 ± 23.70	0.207
Intraventricular hemorrhage [%, (n)]	43.55 (27)	46.63 (173)	0.652
ICH growth [%, (n)]	16.13 (10)	20.49 (76)	0.426
**Comorbidities**			
Hypertension [%, (n)]	30.65 (19)	52.02 (193)	0.002
Diabetes mellitus [%, (n)]	4.84 (3)	17.52 (65)	0.011
Smoking [%, (n)]	3.23 (2)	7.55 (28)	0.215
**Location**			
Frontal lobe [%, (n)]	27.42 (17)	19.95 (74)	0.181
Parietal lobe [%, (n)]	32.26 (20)	21.83 (81)	0.072
Occipital lobe [%, (n)]	3.23 (2)	8.09 (30)	0.176
Temporal lobe [%, (n)]	22.58 (14)	12.94 (48)	0.045
Insular lobe [%, (n)]	0 (0)	0.81 (3)	0.477
Brain stem [%, (n)]	0 (0)	4.04 (15)	0.107
Cerebellum [%, (n)]	24.19 (15)	9.97 (37)	0.001
Basal ganglia [%, (n)]	22.58 (14)	36.93 (137)	0.028
**Shape descriptors**			
Compactness ± SD	0.66 ± 0.22	0.58 ± 0.2	0.005
Circle factor ± SD	0.35 ± 0.15	0.41 ± 0.15	0.002
Fourier factor ± SD	0.82 ± 0.09	0.86 ± 0.07	< 0.001
Fractal dimension ± SD	0.38 ± 0.19	0.42 ± 0.26	0.301
**Histogram analysis**			
Mean ± SD	1.54 ± 0.4	1.68 ± 0.32	0.001
Coefficient of variance ± SD	1.53 ± 0.98	1.47 ± 0.53	0.52
Skewness ± SD	2.53 ± 20.59	−0.92 ± 9.85	0.036
Kurtosis ± SD	1.66 ± 24.56	1.17 ± 22.46	0.875
**Texture analysis**			
Energy ± SD	0.23 ± 1.31	0.04 ± 0.93	0.048
Entropy ± SD	0.18 ± 1.05	0.03 ± 0.99	0.120
Contrast ± SD	0.31 ± 1.21	0.05 ± 0.95	0.008
Homogeneity ± SD	0.32 ± 1.09	0.05 ± 0.97	0.006

*SD* standard deviation, *ICH* intracerebral hemorrhage

**Table 2:** Multivariate logistic regression model of factors associated with 30-day mortality among surgically and conservatively treated patients

*Variable*	*OR*	*95% CI*	*p‑value*
**Logistic regression analysis** **(*n*** **=** **435)**			
Glasgow Coma Scale	0.693	0.613–0.773	< 0.001
Volume	1.029	1.007–1.054	0.012
Glucose level	1.188	1.058–1.353	0.005
Subtentorial location	0.101	0.016–0.511	0.009
Intraventricular hemorrhage	2.597	1.217–5.677	0.015
Fractal dimension	1.179	1.013–1.453	0.102
Energy	1.389	1.19–1.821	0.002
Entropy	0.763	0.598–0.88	0.005
Surgical treatment	0.226	0.049–0.85	0.039
**Moderated logistic regression analysis** **(*n*** **=** **435)**			
Surgical treatment × Glasgow Coma Scale	1.082	0.859–1.294	0.433
Surgical treatment × volume	0.905	0.902–0.908	< 0.001
Surgical treatment × subtentorial location	N/A	N/A	N/A
Surgical treatment × intraventricular hemorrhage	1.154	1.034–1.628	0.010
Surgical treatment × energy	0.932	0.884–0.988	0.010
Surgical treatment × entropy	0.927	0.846–0.991	0.051
Surgical treatment × glucose	1.003	0.663–1.572	0.988

*CI* confidence interval, *OR* odds ratio, *N/A* not-applicable

### Moderation Effect of ICH Energy

**Fig. 2:**
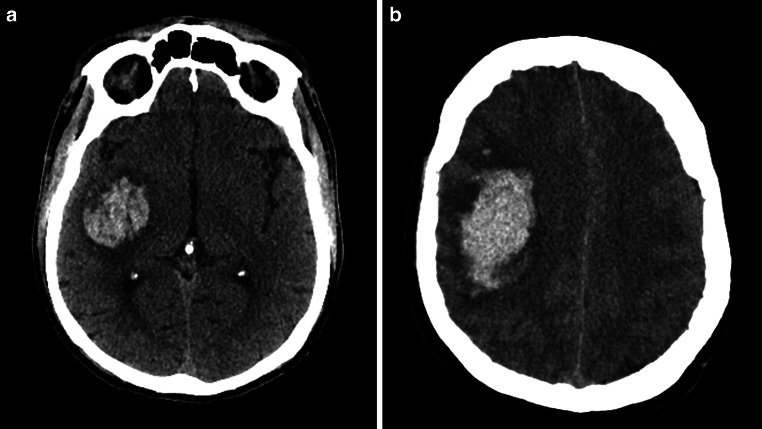
Comparison of ICH with low (**a**) and high (**b**) energy

**Fig. 3:**
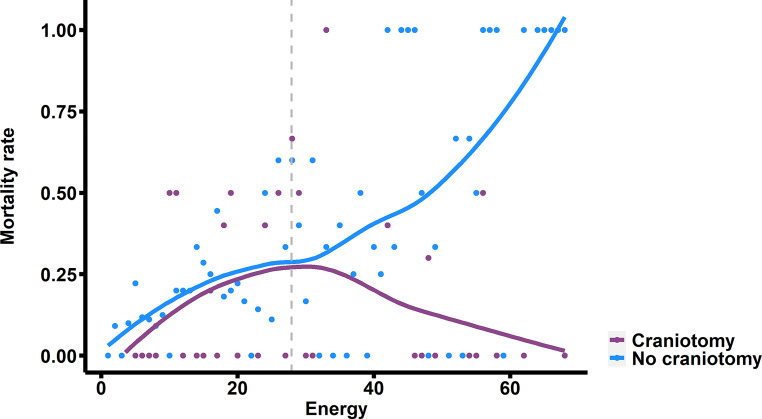
Correlation of mortality rate and ICH energy among patients who underwent surgical and conservative treatment. *Grey dashed line* represents determined energy threshold of 27.92

Our findings indicate a significant positive moderation by ICH energy on the reduction of 30-day mortality following surgical treatment (OR: 0.932; 95% CI: 0.884–0.988; *p* = 0.01) (Table 2). To distinguish between patients with low and high nergye, we established the cut-off point for such moderation at 27.92 (*p* < 0.01). Examples of ICH with high and low energy are presented in Fig. 2. Among patients above this threshold (54.50%) surgical treatment was independently associated with a lower risk of 30-day mortality (OR: 0.113; 95% CI: 0.015–0.593; *p* = 0.02). Additionally, a lower GCS score upon admission (OR: 0.731; 95% CI: 0.632–0.83; *p* < 0.01), a larger volume of ICH (OR: 1.26; 95% CI: 1.021–1.53; *p* = 0.043), higher glucose levels at admission (OR: 1.246; 95% CI: 1.091–1.476; *p* < 0.01), and the presence of IVH (OR: 3.177; 95% CI: 1.366–7.769) were independently associated with an increased risk of 30-day mortality. Below determined threshold (45.50%) only older age (OR: 1.068; 95% CI: 1.025–1.120; *p* < 0.01) and the subtentorial location (OR: 1.020; 95% CI: 1.003–1.041; *p* = 0.031) were independently associated with higher risk of 30-day mortality (Table 3**, **Fig. 3).

**Table 3:** Multivariate logistic regression analysis models of factors associated with 30-day mortality for patients with high and low intracerebral hemorrhage energy

*Variable*	*OR*	*95% CI*	*p‑value*
**High energy** **(*n*** **=** **236)**			
Glasgow Coma Scale	0.731	0.632–0.83	< 0.001
Age	1.012	0.987–1.039	0.342
Volume	1.26	1.021–1.53	0.043
Subtentorial location	0.447	0.082–2.174	0.328
Entropy	0.999	0.998–1.001	0.38
Fractal dimension	1.461	1.123–1.945	0.006
Intraventricular hemorrhage	3.177	1.366–7.769	0.009
Glucose	1.246	1.091–1.476	0.004
Surgical treatment	0.113	0.015–0.593	0.018
**Low Energy** **(*n*** **=** **197)**			
Glasgow Coma Scale	0.995	0.949–1.046	0.852
Age	1.068	1.025–1.120	0.003
Volume	0.080	0.002–1.121	0.100
Subtentorial location	1.020	1.003–1.041	0.031
Entropy	0.947	0.673–1.332	0.744
Fractal dimension	1.689	0.355–7.832	0.497
Intraventricular hemorrhage	0.997	0.709–1.305	0.983
Glucose	0.121	0.007–1.241	0.099
Surgical treatment	0.995	0.949–1.046	0.852

*CI* confidence interval, *OR* odds ration

### Moderation Effect of ICH Volume

**Fig. 4:**
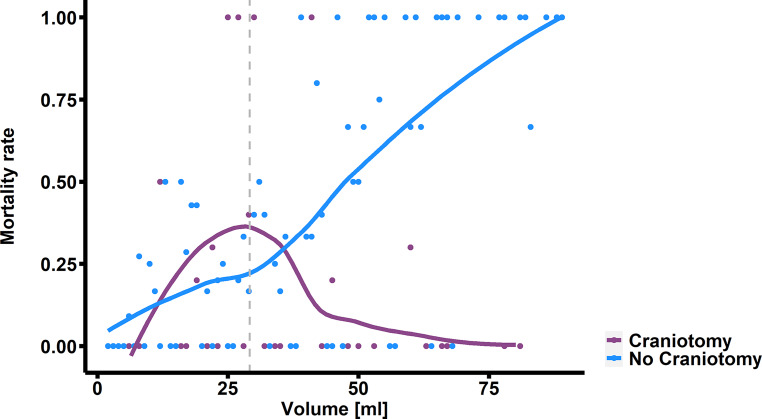
Correlation of mortality rate and ICH volume among patients who underwent surgical and conservative treatment. *Grey dashed line* represents determined energy threshold of 29.47 ml

Furthermore, we found a significant positive moderation by ICH volume on the reduction of 30-day mortality following surgical treatment (OR: 0.905; 95% CI: 0.902–0.908; *p* < 0.01). To distinguish between patients with low and high volumes, we established cut-off point for volume moderation at 29.47 ml (*p* < 0.01). Among patients above that threshold (49.19%), surgical treatment was associated with lower risk of 30-day mortality (OR: 0.037; 95% CI: 0.001–0.414; *p* = 0.03), a higher GCS score upon admission (OR: 0.764; 95% CI: 0.657–0.872; *p* < 0.01) and a higher entropy (OR: 0.994; 95% CI: 0.986–0.998; *p* = 0.04). A higher glucose level upon admission (OR: 1.22; 95% CI: 1.063–1.443; *p* < 0.01), presence of IVH (OR: 3.979; 95% CI: 1.495–11.401; *p* < 0.01) and a higher fractal dimension (OR: 1.489; 95% CI: 1.114–2.091; *p* = 0.01) were independently associated with a higher risk of 30-day mortality among those patients. In terms of patients with lower ICH volume (50.81%) higher GCS score (OR: 0.570; 9%% CI: 0.435–0.695; *p* < 0.01) and subtentorial location (OR: 0.035; 95% CI: 0.001–0.442; *p* = 0.02) were independently associated with lower risk of 30-day mortality and higher energy was associated with higher risk of 30-day mortality (OR: 1.419; 95% CI: 1.175–2.302; *p* = 0.03) (Table 4**, **Fig. 4).

**Table 4:** Multivariate logistic regression analysis models of factors associated with 30-day mortality for patients with high and low intracerebral hemorrhage volume

*Variable*	*OR*	*95% CI*	*p‑value*
**High volume** **(*n*** **=** **213)**			
Glasgow Coma Scale	0.764	0.657–0.872	< 0.001
Age	0.993	0.961–1.025	0.643
Energy	1.376	1.148–1.909	0.010
Subtentorial location	0.185	0.017–1.5	0.135
Entropy	0.994	0.986–0.998	0.037
Fractal dimension	1.489	1.114–2.091	0.013
Intraventricular hemorrhage	3.979	1.495–11.401	0.007
Glucose	1.22	1.063–1.443	0.008
Surgical treatment	0.037	0.001–0.414	0.029
**Low volume** **(*n*** **=** **220)**			
Glasgow Coma Scale	0.570	0.435–0.695	< 0.001
Age	1.028	0.984–1.08	0.240
Energy	1.419	1.175–2.302	0.025
Subtentorial location	0.035	0.001–0.442	0.018
Entropy	0.994	0.982–0.999	0.091
Fractal dimension	0.914	0.587–1.357	0.668
Intraventricular hemorrhage	1.443	0.316–6.634	0.630
Glucose	1.310	0.967–1.781	0.073
Surgical treatment	0.768	0.086–5.588	0.800

*CI* confidence interval, *OR* odds ratio

## Discussion

Our study revealed that the majority of patients received conservative treatment, which is consistent with the commonly adopted management approach [10]. Surgically treated patients exhibited significantly fewer comorbidities, despite being in a worse neurological state. Moreover, surgical treatment independently contributed to a reduction in 30-day mortality. As previously mentioned, although the STICH trial failed to demonstrate the benefits of surgical treatment for of ICH [6], further analysis revealed that this approach may enhance outcomes for certain patient groups [7, 9]. Similarly, our study identified factors that could moderate the impact of ICH removal on mortality, which should be considered in management planning.

We uncovered significant differences in the CT appearance of surgically treated ICH. Based on existing literature, ICH radiomics have not been analyzed for surgical treatment indications; however, it’s association with outcomes and the risk of ICH growth has been demonstrated [11–13]. This aligns with our findings that energy and entropy were independently linked to an increased risk of 30-day mortality in our study group. Another interesting finding in our study was the moderating influence of energy levels on 30-day morality postsurgical treatment. Patients with higher ICH energy levels may benefit more from surgical intervention, which likely stems from an increased risk of ICH expansion [16]. Consequently, early surgical intervention and the application of hemostatic materials could be crucial in prevention of further rebleeding. Interestingly, another study with comparable number of patients found that in both high and low energy groups, well-known outcome predictors like GCS score, glucose level, ICH volume, or presence of IVH [10] were significantly correlated with mortality only in patients exhibiting high ICH energy. This finding might indicate a divergent natural progression of the disease between these two patient groups. Therefore, further research into radiomics in ICH management could aid in clarifying the role of surgical treatment, including the identification of patient subgroups which might distinctly benefit from it.

Our research also identified a significant moderating role of volume in the connection between 30-day mortality and surgical intervention. Following an ICH removal, a significant reduction in 30-day mortality was observed only in patients with larger ICH volumes. As previously documented in the literature, ICH size is one of the most important predictors of outcome [17]. Larger ICHs are associated with a mass effect, potentially leading to brain herniation. Additionally, a greater volume of blood can amplify its toxic impact on brain tissue [18] and is linked to an increased risk of ICH growth and early deterioration [19]. Our findings are consistent with other researchers who have also proposed that larger ICHs are an indication for surgical intervention [4, 5]. Other research groups also found that patients with such ICHs are more frequently candidates for surgery [20]. In contrast to ICH energy, common risk factors had similar impacts on mortality both in groups with larger and smaller ICH.

Moreover, we identified a moderating negative impact of IVH on mortality after surgical treatment. This is consistent with previous studies, indicating that patients with IVH did not benefit from surgical interventions [6, 21]. Presence of IVH has been previously established as independent factor for increased mortality after ICH [22]. Furthermore, it has been observed that patients with IVH are less likely to undergo surgery [20].

Our study highlights the value of radiomics analysis in aiding clinical decision-making for the treatment of patients with spontaneous ICH. We found that patients with higher volume and energy of ICH might benefit from surgical treatment, although its effectiveness could be diminished in cases with intraventricular blood; however, our study encountered certain limitations. Firstly, it was nonrandomized, and due to the absence of definitive guidelines for surgical treatment, decisions were based on clinical experience, potentially introducing bias. Another limitation is its single-center, retrospective nature. Hence, a larger multicenter randomized control trial is warranted to establish comprehensive ICH management guidelines that incorporate radiomic analysis. Despite these constraints, our study is pioneering in demonstrating the role of radiomics in influencing the outcomes of surgical treatment.

### Supplementary Information

Supplemental Methods - details about image segmentation and paramters calculation; Supplementary Table - Additional comparison of patients treated surgically and conservatively.
