# Supplementary material for: Association of Imaging-based Predictors with Outcome in Different Treatment Options for Intracerebral Hemorrhage
Source: Clin Neuroradiol. 2024 Apr 26;34(3):685–92. doi: 10.1007/s00062-024-01406-2 (PMC11339125; doi:10.1007/s00062-024-01406-2)
Supplement: Supplementary file 1 — Supplemental Methods - details about image segmentation and paramters calculation; Supplementary Table - Additional comparison of patients treated surgically and conservatively. [file 62_2024_1406_MOESM1_ESM.docx]

Supplementary Information

Title: Association of imaging-based predictors with outcome in different treatment options for intracerebral haemorrhage.

# Supplemental Methods

## *CT image segmentation*

All analyses were performed using software of our own design, written in Python 3.8 with OpenCV library. To analyse ICH appearance on CT we performed series of transformations on each slice of head CT scans obtained from patients. First, histogram of each image was equalized to increase its contrast. Then, we applied Gaussian blur filter to reduce noise. ICH was selected manually with mouse click inside its area. Then, images were binarized and contour extraction was performed. Contour closest to manually selected point was determined as ICH contour and used for further analysis. From non-transformed CT images we obtained all pixels within selected contour – ICH image. Then, each point of contour was moved 4 mm along vector between that point and original contour centroid. Pixels between original and enlarged contour was then extracted as perihaemorrhage area (PHA) image. To obtain reference image, point from corresponding brain area on side contralateral to ICH location was manually selected. We then obtained 20x20 pixel rectangular image surrounding such point and designated it as healthy brain tissue sample.

## *Contour analysis*

To analyse ICH contour, similarly as in our previous articles, we calculated four shape descriptors: Compactness (C), Fractal Dimension (FD), Fourier Factor (FF) and Circle Factor (CF). C is the efficiency for the contour to contain a given area. It is defined with formula:

$$C=1- \frac{4\pi A}{p^{2}}$$

Where *p* is perimeter of the contour and *A* is its enclosed area.

To calculate FD, we used units of different length to measure contour perimeter. Then, we fitted linear regression model to graph of *log p*, where *p* is the contour perimeter, against the corresponding values of *log (1/s)*, where *s* is length of unit. FD is slope of such model.

FF was obtained from following formula:

$$FF=1- \frac{\sum_{k=-\frac{N}{2}+1}^{N/2} (\frac{\left| Z_{0}\left( k \right) \right|}{\left| k \right|})}{\sum_{k=-\frac{N}{2}+1}^{N/2} \left| Z_{0}(k) \right|}$$

where *k* = 0, …, N -1, N is the number of pixels representing contour and *Z_0_(k*) are normalized Fourier descriptors, calculated with Fast Fourier Transform:

$$Z\left( k \right)=DFT\left( z\left( n \right) \right)= \frac{1}{N}\sum_{n=0}^{N-1} z(n)e^{-i2\pi nk/N}$$

Fourth descriptor, CF, was defined with formula:

$$CF= \frac{p}{p_{c}}$$

Where *p* is ICH contour perimeter and *p_c_* is perimeter of largest circle that can be inscribed into ICH contour.

Values of all descriptors used for analysis were means of descriptors calculated for each CT slice with visible ICH.

## *Density distribution analysis*

Extracted ICH, PHA and brain tissue images were presented as arrays of density values (varying from 0 to 255). For each image we analysed four moments of such density values distribution: mean, coefficient of variance (defined as mean/standard deviation), skewness and kurtosis. Values of all descriptors used for analysis were also means of descriptors calculated for each CT slice with visible ICH.

## *Texture analysis*

Texture analysis was performed for ICH, PHA and brain tissue image. First, we quantized density values into 16 value levels. Then we constructed co-occurrence matrices (matrices with distribution of co-occurring pixel values at a given offset)[]:

$$M_{\Delta x, \Delta y}\left( i, j \right)= \sum_{x=1}^{n} \sum_{y=1}^{m} \left\{ \begin{aligned} 1, if I\left( x, y \right)=i and I\left( x+ \Delta x, y+ \Delta y \right)=j \\ 0, otherwise \end{aligned} \right.$$

where *i* and *j* are pixel values, *x* and *y* are the spatial positions in the *n x m* size image *I* and *(Δx, Δy)* is the given offset. For each image we constructed four matrices with offset d = (2,2) in directions of 0, 45, 90 and 135 degrees. Based on matrices we calculated following descriptors:

Energy = $\sqrt{\sum_{i,j=0}^{V-1} {(M\left( i, j \right))}^{2}}$

Homogeneity = $\sum_{i,j=0}^{V-1} \frac{M(i,j)}{1+ {(i+j)}^{2}}$

Correlation = $\sum_{i,j=0}^{V-1} M\left( i,j \right)\left[ \frac{(i- \bar{i})(j- \bar{j})}{\sqrt{\left( \sigma_{i}^{2} \right)(\sigma_{j}^{2})}} \right]$

Contrast = $\sum_{i,j=0}^{V-1} {(i-j)}^{2} M(i,j)$

where *V* is the number of value levels (in our analysis *V* = 16). Values of abovementioned features are means of values calculated from four matrices and each CT slice with visible ICH.

Supplementary Table 1. Additional comparison of patients treated surgically and conservatively.

| **Variable** | **Surgical treatment**  **(n = 62)** | **Conservative treatment**  **(n = 373)** | **p-value** |
| --- | --- | --- | --- |
| **Comorbidities** | | | |
| History of subarachnoid hemorrhage [%] | 1.61 (1) | 0.81 (3) | 0.540 |
| History of ischemic stroke [%] | 4.84 (3) | 12.94 (48) | 0.067 |
| Atrial fibrillation [%] | 11.29 (7) | 20.49 (76) | 0.089 |
| Ischemic heart disease [%] | 1.61 (1) | 14.56 (54) | 0.005 |
| Renal diseases [%] | 3.23 (2) | 9.16 (34) | 0.117 |
| Lungs diseases [%] | 0 (0) | 5.39 (20) | 0.061 |
| **Current medications** | | | |
| Acetylsalicylic acid [%] | 6.45 (4) | 16.98 (63) | 0.034 |
| Β-blockers [%] | 11.29 (7) | 25.88 (96) | 0.013 |
| AT_2_-blockers [%] | 0 (0) | 4.31 (16) | 0.096 |
| Calcium channel blockers [%] | 9.68 (6) | 9.97 (37) | 0.943 |
| ACEI [%] | 8.06 (5) | 19.95 (74) | 0.025 |
| Diuretics [%] | 14.52 (9) | 21.02 (78) | 0.236 |
| Steroids [%] | 0 (0) | 0.54 (2) | 0.562 |
| Hypoglycemic medications [%] | 3.23 (2) | 11.05 (41) | 0.057 |
| Anticoagulants [%] | 11.29 (7) | 16.71 (62) | 0.28 |
| Antiplatelet therapy [%] | 1.61 (1) | 7.01 (26) | 0.104 |
| Nitrates [%] | 0 (0) | 0.27 (1) | 0.682 |
| Statins [%] | 4.84 (3) | 16.17 (60) | 0.019 |
| **Laboratory tests results** | | | |
| Red Blood Cells count [10^3^/μl] ± SD | 4.16 ± 0.73 | 4.43 ± 0.62 | 0.007 |
| White Blood Cells count [10^3^/μl] ± SD | 11.26 ± 5.04 | 10.59 ± 7.36 | 0.551 |
| Haemoglobin [g/dl] ± SD | 12.56 ± 2.18 | 13.27 ± 1.89 | 0.019 |
| Mean Corpuscular Volume [μm^3^] ± SD | 89.60 ± 6.67 | 88.56 ± 5.42 | 0.237 |
| Mean Corpuscular Haemoglobin [pg] ± SD | 30.37 ± 2.70 | 30.02 ± 2.15 | 0.327 |
| Mean Corpuscular Haemoglobin Concentration [g/dl] ± SD | 33.87 ± 1.43 | 33.9 ± 1.19 | 0.896 |
| Hematocrit [%] ± SD | 37.02 ± 5.76 | 39.11 ± 5.11 | 0.011 |
| Platelet count [103/μl] ± SD | 213.3 ± 91.45 | 213.73 ± 77.33 | 0.972 |
| Glucose [mmol/l] ± SD | 6.34 ± 2.30 | 7.07 ± 2.86 | 0.147 |
| Creatinine [μmol/l] ± SD | 71.71 ± 22.05 | 89.91 ± 52.05 | 0.032 |
| Urea [mmol/l] ± SD | 6.15 ± 3.71 | 6.75 ± 3.83 | 0.357 |
| Sodium [mmol/l] ± SD | 140.93 ± 5.06 | 140.1 ± 4.93 | 0.322 |
| Potassium [mmol/l] ± SD | 3.98 ± 0.48 | 3.99 ± 0.55 | 0.885 |
| Activated Partial Thromboplastin Time [s] ± SD | 29.02 ± 5.00 | 30.99 ± 8.09 | 0.199 |
| International Normalized Ratio ± SD | 1.11 ± 0.30 | 1.16 ± 0.49 | 0.576 |
| CRP | 56.32 ± 69.17 | 27.38 ± 47.04 | 0.015 |
| **Histogram analysis of perihemorrhage area** | | | |
| Perihemorrhage area mean ± SD | 0.94 ± 0.25 | 0.98 ± 0.18 | 0.142 |
| Perihemorrhage area coefficient of variance ± SD | 2.29 ± 1.78 | 1.95 ± 1.44 | 0.102 |
| Perihemorrhage area skewness ± SD | 4.27 ± 26.41 | 0.55 ± 10.69 | 0.054 |
| Perihemorrhage area kurtosis ± SD | 6.81 ± 42.29 | -3.4 ± 67.3 | 0.248 |
| **Texture analysis of perihemorrhage area** | | | |
| Perihemorrhage area energy ± SD | 0.26 ± 1.16 | 0.04 ± 0.97 | 0.028 |
| Perihemorrhage area entropy ± SD | 0.3 ± 1.17 | 0.05 ± 0.96 | 0.011 |
| Perihemorrhage area contrast ± SD | -0.26 ± 0.92 | 0.04 ± 1.01 | 0.026 |
| Perihemorrhage area homogeneity ± SD | 0.13 ± 1.13 | 0.02 ± 0.98 | 0.255 |
